# Suppression of Foxo1 Activity and Down-Modulation of CD62L (L-Selectin) in HIV-1 Infected Resting CD4 T Cells

**DOI:** 10.1371/journal.pone.0110719

**Published:** 2014-10-16

**Authors:** Benjamin Trinité, Chi N. Chan, Caroline S. Lee, Saurabh Mahajan, Yang Luo, Mark A. Muesing, Joy M. Folkvord, Michael Pham, Elizabeth Connick, David N. Levy

**Affiliations:** 1 Department of Basic Science, New York University College of Dentistry, New York, New York, United States of America; 2 Aaron Diamond AIDS Research Center, New York, New York, United States of America; 3 Department of Medicine, University of Colorado Denver, Aurora, Colorado, United States of America; George Mason University, United States of America

## Abstract

HIV-1 hijacks and disrupts many processes in the cells it infects in order to suppress antiviral immunity and to facilitate its replication. Resting CD4 T cells are important early targets of HIV-1 infection in which HIV-1 must overcome intrinsic barriers to viral replication. Although resting CD4 T cells are refractory to infection *in vitro*, local environmental factors within lymphoid and mucosal tissues such as cytokines facilitate viral replication while maintaining the resting state. These factors can be utilized *in vitro* to study HIV-1 replication in resting CD4 T cells. *In vivo*, the migration of resting naïve and central memory T cells into lymphoid tissues is dependent upon expression of CD62L (L-selectin), a receptor that is subsequently down-modulated following T cell activation. CD62L gene transcription is maintained in resting T cells by Foxo1 and KLF2, transcription factors that maintain T cell quiescence and which regulate additional cellular processes including survival, migration, and differentiation. Here we report that HIV-1 down-modulates CD62L in productively infected naïve and memory resting CD4 T cells while suppressing Foxo1 activity and the expression of KLF2 mRNA. Partial T cell activation was further evident as an increase in CD69 expression. Several other Foxo1- and KLF2-regulated mRNA were increased or decreased in productively infected CD4 T cells, including IL-7rα, Myc, CCR5, Fam65b, S1P_1_ (EDG1), CD52, Cyclin D2 and p21^CIP1^, indicating a profound reprogramming of these cells. The Foxo1 inhibitor AS1842856 accelerated *de novo* viral gene expression and the sequella of infection, supporting the notion that HIV-1 suppression of Foxo1 activity may be a strategy to promote replication in resting CD4 T cells. As Foxo1 is an investigative cancer therapy target, the development of Foxo1 interventions may assist the quest to specifically suppress or activate HIV-1 replication *in vivo*.

## Introduction

Resting CD4+ T cells in lymphoid tissues and mucosa constitute at least 90% of HIV/SIV RNA+ cells [1]–[5] and are massively depleted during acute infection, [2]–[6], a period in which antiviral immunity is established and the outcome of infection determined [7]. Although peripheral blood CD4+ T cells are resistant to infection *in vitro* [8]–[10], viral replication occurs predominantly in lymphoid and mucosal tissues, where microenvironmental factors, including cytokines such as IL-4 and IL-7 maintain resting T cell viability [11]–[17], and fortuitously (for the virus), facilitate their productive infection [18]–[22]. IL-7 is the primary cytokine maintaining the survival and homeostasis of mature T cells [17], [23], [24]. In addition, HIV-1 can itself manipulate infected cells in order to enhance viral replication; for example, binding of viral envelope proteins to CD4 and coreceptors CXCR4 and CCR5 can stimulate actin remodeling to facilitate early replication steps in resting T cells [25]. Viral proteins such as Env, Tat and Nef interact with cell signaling pathways and can stimulate partial T cell activation that enhances virus expression in resting T cells [26]–[29].

An important revelation of the past decade has been that T cell quiescence results not merely from an absence of antigenic stimulation, but is actively maintained by constitutive expression of certain transcription factors [30]–[32]. Chief among these is Foxo1, a member of the Forkhead box protein family of proteins, (the other members being Foxo3a, Foxo4 and Foxo6) that respond to environmental stimuli (growth factors, oxidative stress, nutritional availability) and regulate the expression of many genes involved in cell growth, proliferation, differentiation, and survival [33]–[37]. Foxo3a has been implicated in HIV-1 neuropathology [38] and disease progression [39], [40]. Foxo1 is specifically upregulated during T cell maturation [41] and is constitutively active in resting T cells [35], [42] and many other tissues [43]. Deletion of Foxo1 leads to spontaneous T cell activation and differentiation [44], while ectopic expression of constitutively active Foxo1 suppresses T cell proliferation [36], [45]. Foxo1 binds directly to a consensus DNA sequence on promoters that it regulates [35], [46] and it also associates with many binding partners [47] to regulate gene expression. Foxo1 transactivates and maintains expression of CD62L, CCR7, KLF2 (LKLF) [45] and the IL-7 receptor alpha chain (IL-7rα) in resting T cells. Transcriptional repression of these genes is an indicator of Foxo1 inactivation and early T cell activation [45], [48]–[50]. Thus, by controlling these genes, Foxo1 regulates both T cell quiescence and T cell trafficking [33], [48], [51].

CD62L is expressed on resting naïve, central memory and some effector memory T cells and regulates their migration into lymph nodes [52]. Disruption of CD62L expression has detrimental effects on T cell migration and immune responses [35], [53], [54]. Antigenic activation in the LN leads first to rapid CD62L shedding by protease cleavage and then to transcriptional suppression [55] as a result of Foxo1 inactivation [33]. CD62L down-modulation functions to prevent activated and CD62L-negative effector memory T cells from re-entering LN. KLF2, often cooperatively with Foxo1, also transactivates CD62L [56] as well as a further set of genes involved in cell growth, differentiation, and migration [57]–[61].

In this study we examine the consequences of HIV-1 infection to naïve and memory resting CD4+ T cells, finding that CD62L was specifically down-modulated, the early activation marker CD69 was upregulated, and that these occurred concomitantly with HIV-1 suppression of Foxo1 activity. Several genes that are known targets of Foxo1 and KLF2 were activated or repressed in infected resting T cells [34], [45], [62]–[64], including IL-7 receptor (IL-7rα), Myc, S1P_1_ (EGD1), CD52, CCR5, Fam65b, Cyclin D2 and p21^CIP1^. Each of these genes regulates cell survival, differentiation, activation and/or migration. Application of the Foxo1 inhibitor AS1842856 resulted in an acceleration of HIV-1 replication in resting CD4+ T cells, suggesting that targeting Foxo1 may be a viral tactic to promote its own replication.

## Results

### HIV-1 infection of resting CD4+ T cells leads to down-modulation of CD62L and upregulation of CD69

We first tested whether common gamma chain cytokines alter CD62L expression on resting naïve and memory peripheral blood CD4 T cells, finding that each maintained high CD62L expression at concentrations which enhance HIV-1 infection (Figure S1 in File S1). Because of the demonstrated importance for IL-7 in maintaining T cell homeostasis *in vivo* [23], we chose this cytokine for the majority of the following studies. Maintenance of CD62L expression in IL-7 culture is consistent with the finding that submitogenic levels of IL-7 do not activate PI3K [65].

**Figure 1:**
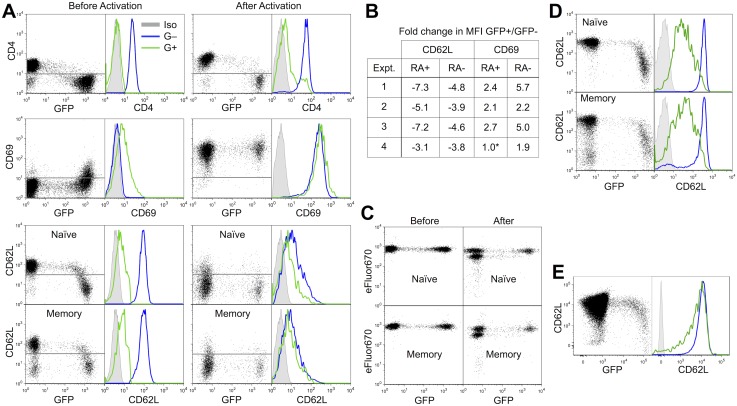
Down-modulation of CD62L and upregulation of CD69 and on productively infected naïve and memory resting CD4 T cells. **A.** Resting IL-7 treated CD4+ T cells were infected with the GFP reporter virus NLENG1, restricted to a single round of infection by treatment with the protease inhibitor indinavir (2 µM). Flow analysis was carried out on day 5 post infection on cells with and without activation by αCD3/CD28 beads on day 3 p.i. Grey: isotype-matched IgG control antibody staining. Blue: GFP-negative cells. Green: GFP+ cells. Data are representative of 4 independent experiments. **B.** Fold change in CD62L and CD69 expression on naïve and memory CD4+ resting T cells in A. With on exception (*) all values were statistically different (p<0.01) from the negative control by a T(x) population comparison test [134]. **C.** Failure of GFP+ cells proliferate demonstrates that CD62L-low cells are not produced by preferential expansion of an existing CD62L-low population. Experiment shown is representative of >5 similar experiments. **D.** IL-4 supports HIV-1-induced down-modulation of CD62L on both naïve and memory resting CD4+ T cells. Experiment shown is representative of >3 similar experiments. **E.** CD62L down-modulation on HIV-1 infected naïve tonsil T cells 7 days after infection. Similar results were observed on CD45R0+cells (not shown). Experiment shown is representative of 2 similar experiments.

We next examined the influence of HIV-1 infection on expression of CD4, CD69 and CD62L on resting CD4+ T cells (Figure 1). We activated a subset of cells with anti-CD3/CD28 beads 2 days after infection in order to compare receptor expression on infected resting vs. activated cells. CD4 was efficiently down-modulated by the virus, as expected, whether the cells were activated or not after infection (Figure 1A). Interestingly, the early activation marker CD69 was modestly upregulated in both memory (CD4+CD45RA+) and naïve (CD4+CD45RA−) resting GFP+ cells, (Figure 1B). There was variability in the effect of infection on both receptors among cell donors, but with a single exception, all infections produced significant CD62L down modulation and CD69 upregulation. Late activation markers such as CD25, CD38 and HLA Class II were unaffected (not shown). Mock infected cells remained uniformly CD69-negative (not shown), thus HIV-1 is actively inducing CD69 expression.

CD62L was strongly down-modulated in both the naïve (T_N_) and memory subsets (Figure 1A–B), becoming progressively lower as GFP intensity increased past an apparent threshold level of gene expression. After 48 hours of activation with anti-CD3/CD28 beads, which down-modulates CD62L, the GFP+ cells expressed even less CD62L than GFP-negative activated cells. A prior study of pre-activated cells reported HIV-1-induced CD62L down-modulation [66], which these data confirm, despite the natural down-modulation provided by stimulation through the T cell receptor. We further observed that CD62L was down-modulated by HIV-1 on both T_CM_ and T_EM_ subsets identified by CCR7 expression (Figure S2A in File S1). While TN are uniformly CD62L+, a subset of T_EM_ naturally do not express CD62L, complicating interpretation of the data. However, the overall shape of the relationship between GFP levels and CD62L levels provides evidence that HIV-1 is indeed down-modulating CD62L on those cells which did express this receptor prior to infection. CCR7 was modestly down-modulated in GFP+ resting naïve and memory cells (Figure S2B in File S1). This is consistent with a recent study in activated T cells, where CCR7 was down-modulated by HIV-1 Vpu [67]. Interestingly, HIV-1 expression was considerably higher in T_EM_ than in either T_N_ or T_CM_ (Figure S2C in File S1). To test whether a pre-existing CD62L-negative subpopulation was being preferentially expanded after infection, as opposed to HIV-1 actively down-modulating CD62L, we examined cell replication by dilution of the intracellular dye eFluor670 (Figure 1C). Whether left resting or when activated with beads, the population of GFP+ cells did not expand, held in check by Vpr [68] in this post-infection activation scenario [22]. Therefore, CD62L down-modulation appears to be driven by *de novo* HIV-1 expression in these cells.

IL-4 is a product of activated T cells present in lymphoid tissues, including tonsils, where it enhances HIV-1 infection [69]. We have utilized this cytokine to provide a convenient *in vitro* model for HIV-1 infection and latency after direct infection of resting CD4+ T cells [22]. Because of its favorable characteristic of inducing little homeostatic proliferation while efficiently enhancing HIV-1 infection [22], IL-4 is a useful alternative to IL-7 in such studies. We examined CD62L in IL-4 treated resting peripheral blood CD4+ T cells, finding similar down-modulation as in IL-7 treated cells (Figure 1D). Next, we examined infection of tonsil cells cultured with IL-4, finding that CD62L was reduced in these cells as well, though not as strongly as in peripheral blood cells. The difference may be that in our infection of tonsil cells the virus was not held to a single round of infection but was allowed to spread within the target cell population. In this expanding infection, many cells are likely to have been infected too recently to fully down-modulate CD62L.

### CD62L down-modulation is reduced by PI3K inhibition

**Figure 2:**
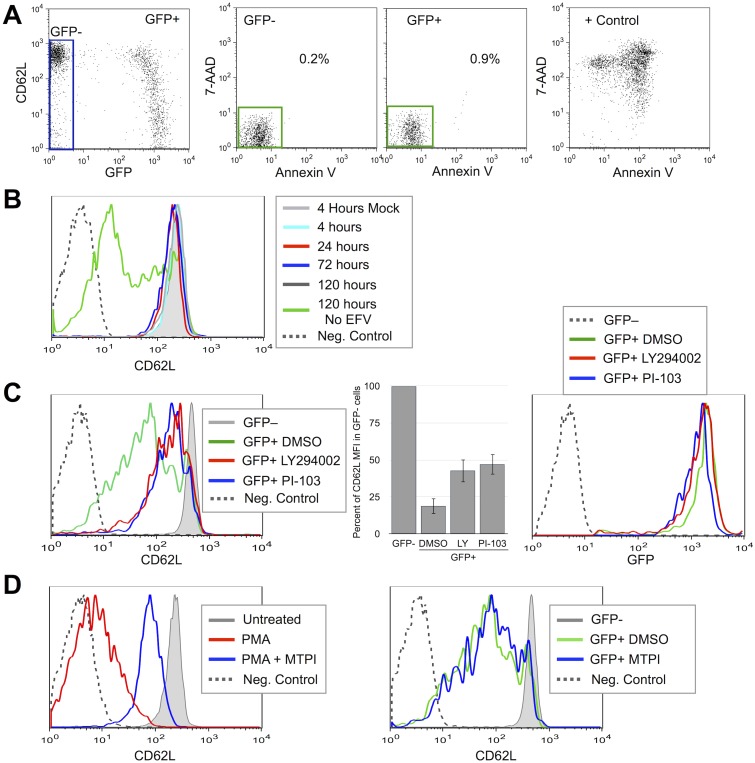
CD62L down-modulation is not the result of apoptosis, virus contact or protease cleavage, but is influenced by PI3K. **A.** Cells infected with for 5 days, then were analyzed for GFP, CD62L and Annexin V surface expression, and permeability to 7-AAD. Data are representative of >5 independent experiments. **B.** Contact with HIV-1 virions does not affect CD62L surface expression. Cells were treated with the reverse transcriptase inhibitor efavirenz (EFV) (2 µg/ml) and infected by spinoculation or spinoculated without virus (Mock). Surface CD62L expression was analyzed at the indicated times after infection. A positive control infection was performed without EFV as indicated. EFV completely prevented the appearance of GFP+ cells (not shown). **C.** The PI3K and mTORC2 inhibitors LY294002 and PI-103 suppress HIV-1-induced CD62L down-modulation. Infected cells were treated with LY294002 (5 µM), PI-103 (5 µM) or a carrier control (DMSO) 1 day and again 4 hours prior to staining for CD62L. Middle graph: Data from 3 independent experiments with different cell donors. Y axis = the mean fluorescence intensity of CD62L staining (MFI) for each condition expressed as a percentage of the CD62L MFI of the GFP-negative cells. Right: GFP expression is modestly suppressed by LY294002 and PI-103 (MFI = 1761 and 1471 respectively, vs.1981 for DMSO control). LY294002 and PI-103 treatments were statistically different (p<0.01) from their respective DMSO controls by a T(x) population comparison test [134]. **D.** Metalloprotease inhibitor (MTPI) TAPI-1 (50 µM) does not inhibit HIV-1-induced CD62L surface down-modulation. Left: Control experiment demonstrating inhibition of PMA-induced CD62L downmodulation by MTPI. Cells were treated with PMA with and without MTPI and analyzed one hour later. Right: Cells infected with HIV-1 GFP reporter virus were treated with MTPI 1 day and again 3 hours prior to staining. Data are representative of 2 independent experiments.

To explore the mechanism(s) responsible for HIV-1-induced CD62L down-modulation, we first tested whether apoptosis of GFP+ cells was inducing CD62L shedding [70], [71]. However, Annexin V and 7-AAD staining were very low (0.9%) on GFP+ cells that were down-modulating or had down-modulated CD62L (Figure 2A). Prior studies have reported that HIV-1 binding to cells can induce ADAM17-dependent shedding of CD62L through the interaction between envelope protein and CD4 or CXCR4 [72], [73], while another study reported upregulation [74]. To test whether virus binding influenced CD62L expression in our system, we stained cells soon after spinoculation of virus onto cells. Infection was performed in the presence of the reverse transcriptase inhibitor efavirenz (EFV) in order to block events downstream of virus binding and entry. No effect on CD62L expression was observed at any time from 4 hours to 5 days after infection in the presence of EFV (Figure 2B). It has also been reported that contact between Jurkat T cells infected with an Envelope wild type virus and uninfected primary cells led to CD62L shedding [72], but in a separate test we observed no CD62L loss by this method either (data not shown). Failure of coculture of infected and uninfected cells to affect CD62L expression is consistent with the results in Figure 1A that CD62L down-modulation was restricted to the productively infected GFP+ cells and was not observed on GFP-negative bystander cells.

CD62L shedding and transcriptional repression can be triggered by a PI3K/Akt-dependent pathway, and this can be inhibited by the PI3K inhibitors LY294002 or PI-103. These inhibitors reduced the HIV-1-induced down-modulation of CD62L (Figure 2C), confirming that at least part of HIV-1′s effect on CD62L expression is through activation of PI3K/Akt pathway. PI3K inhibition can block HIV-1 infection when added early [75], [76], so we added them after the appearance of GFP+ cells but before CD62L down modulation was evident. The timing of addiction to culture may have been too late to completely block CD62L down modulation by HIV-1. We next directly tested whether a metalloprotease was responsible for CD62L loss by applying the metalloprotease inhibitor (MTPI) TAPI-1 to cells. A control experiment utilizing PMA-induced CD62L shedding demonstrated the effectiveness of MTPI to block this strong stimulus (Figure 2D left panel). On the other hand, MTPI had no effect on HIV-1-induced CD62L down-modulation, indicating that in these cells CD62L is being lost from the cell surface by a mechanism other than MTP cleavage.

### Foxo1- and KLF2-regulated mRNA levels in HIV-1 infected resting CD4+ T cells

**Figure 3:**
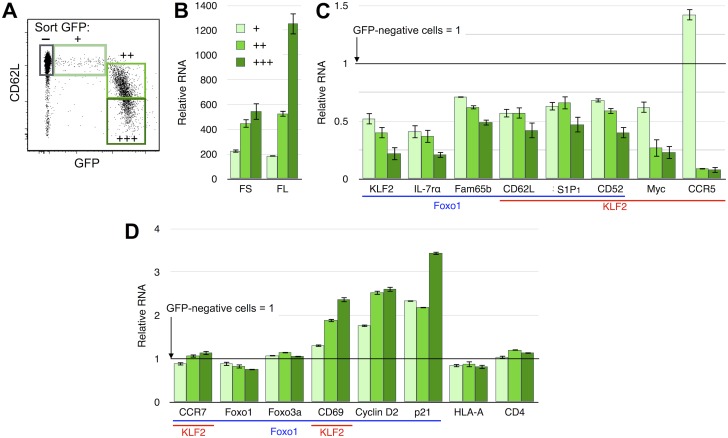
mRNA levels of several known Foxo1- and KLF2-regulated genes in HIV-1 infected naïve CD4+ T cells. Data are representative of 3 independent experiments. Error bars represent SD of triplicate PCR reactions. **A.** CD45RO-CD4+ T cells were infected with sing-round GFP reporter virus for 7 days then sorted by FACS into 4 populations of GFP- and GFP+ cells according to GFP and CD62L expression. **B.** HIV-1 early fully spliced RNA (FS) and late full length RNA (FL) in each group of sorted cells. Data are normalized to a value of 1 for the FS RNA in GFP- cells. GFP- FL RNA was 1.5. A synthetic RNA standard containing both the FS and FL targets was used in order to allow the FS and FL values to be directly compared. Values are presented in Table S2 in File S1. **C.** mRNA of genes that were suppressed in GFP+ cells. Values are normalized to the GFP- cells set to a value of 1 for each gene. Genes regulated by Foxo1 or KLF2 are indicated by color-matched under lines. All p<0.01 (paired T-test). **D.** mRNA of genes that were changed <2 fold or were increased in GFP+ cells. mRNA in GFP-negative cells is set to a relative value of 1 for each gene as in C.

We hypothesized that CD62L transcription was being suppressed in the productively infected cells and that we would observe alterations in the transcription of this and other Foxo1 and KLF2 regulated genes. To test this, we sorted naïve CD4+ T cells with varying degrees of GFP and CD62L expression (Figure 3A) and performed qRT-PCR on their RNA (Figure 3B–C, Table S2 in File S1). We chose to purify naïve cells because they uniformly express CD62L and thus would provide a more consistent test of CD62L mRNA regulation. The comparison between GFP+ and GFP− cells allows us to specifically isolate the effects of HIV-1 gene expression within these cells. We first confirmed that HIV-1 RNA levels corresponded to GFP intensity [22], [77]. Consistent with our hypothesis, CD62L RNA decreased in concordance with increasing HIV-1 expression and decreasing CD62L surface expression. KLF2 mRNA was strongly reduced in progression with HIV-1 and CD62L expression, as were several other Foxo1-regulated mRNA, including IL-7rα, and Fam65B. The KLF2- and Foxo1-regulated S1P_1_, Myc, and CD52 mRNA [62] were all suppressed, and the KLF2-regulated CCR5 mRNA [61] was lower in GFP+ bright cells. Interestingly, CCR5 RNA was modestly increased in the GFP+ dim cells in 2 independent experiments for reasons that are not clear. p21^CIP1^ mRNA was also increased in all productively infected cells. CD69 and Cyclin D2 are negatively regulated by Foxo1, and their RNA increased in progression with HIV-1 expression. Interestingly, some other known targets of Foxo1 transcriptional regulation were not altered in productively infected cells, including CCR7, Foxo3a and Foxo1 itself. The abundances of these mRNA were similar to the starting abundances of the other genes examined (CD62L, etc.) (not shown), so the absence of regulation was genuine and not the result being transcriptionally inactivated prior to infection. Transcriptional regulation is highly complex and cell-type dependent, and since much prior work on these genes has been performed in transgenic mice and in transformed cells *in vitro.* It may not be surprising that some known Foxo1/KLF2 targets genes do not fit the expected pattern in this primary human cell system. Finally, CD4 and HLA-A, genes that are not thought to be Foxo1 or KLF2 targets but which are upregulated in activated T cells, remained unaltered in productively infected cells. This demonstrates that the effect of HIV-1 on T cell activation phenotype is selective and apparently restricted to early activation markers such as CD69.

### Foxo1 relocalizes to the cytoplasm in naïve and memory HIV-1 infected resting CD4+ T cells

**Figure 4:**
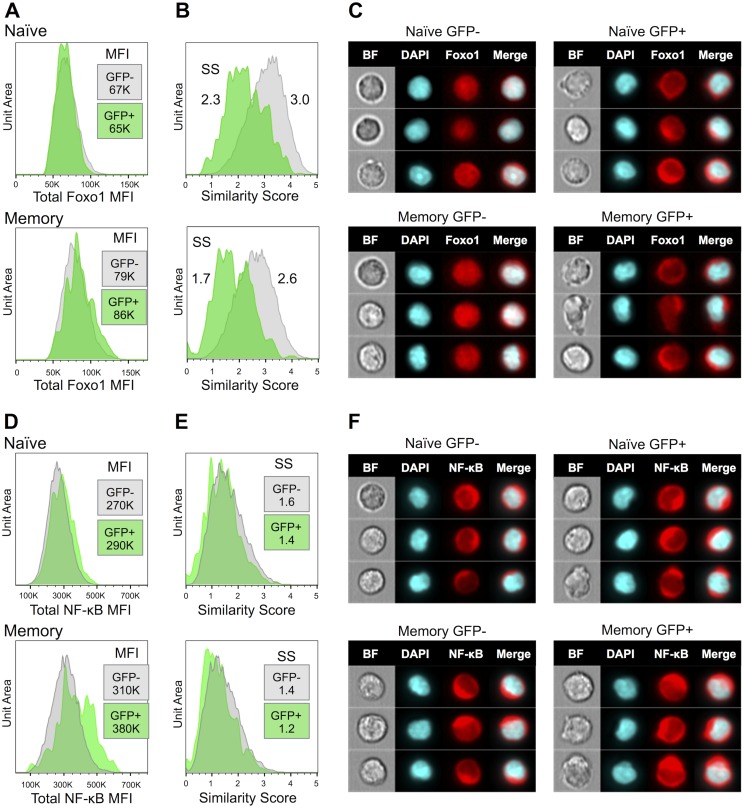
ImageStream analysis of Foxo1 and NF-κB in HIV-1 infected cells. HIV-1 GFP-reporter virus-infected resting CD4+ T cells were stained for CD45RA, intracellular Foxo1 or NF-κB and for DNA (DAPI) 7 days after infection, then analyzed by ImageStream. One representative experiment of two is shown. **A.** Total Foxo1 expression in naïve and memory GFP+ and GFP- cells. MFI = Mean Fluorescence Intensity. **B.** Nuclear localization was analyzed in IDEAS software by comparison of Foxo1 distribution to nuclear DAPI staining using the Similarity Score (see materials and methods). **C.** Representative ImageStream pictures of individual cells stained for Foxo1. **D.** Total NF-κB expression in naïve and memory GFP+ and GFP- cells. **E.** NF-κB nuclear Similarity Scores. **F.** Representative ImageStream pictures of individual cells stained for NF-κB.

Freed from DNA binding, inactivated Foxo1 relocalizes to the cytoplasm; thus cytoplasmic translocation is a direct indication of Foxo1 inactivation. We next examined Foxo1 localization by ImageStream analysis, a technique that combines flow cytometry with cell imaging to provide spatial and intensity information within individual cells (Figure 4). We first observed that total Foxo1 levels were not altered in the productively infected naïve CD4 T cells, and slightly increased in memory cells (Figure 4A). When nuclear and cytoplasmic expression were analyzed, translocation of Foxo1 from nucleus to the (very thin) cytoplasm, was observed in both the naïve and memory resting T cells (Figure 4B). This translocation was clearly evident in images of individual cells (Figure 4C). By contrast with Foxo1, NF-κB remained almost completely cytoplasmic (Figure 4D–F). However, similar to Foxo1, NF-κB total levels were increased in the GFP+ memory cells (Figure 4D), though no nuclear translocation was observed. GFP+ cells tended to be more elongated and irregular in shape, resembling polarized T cells, a further indication of partial activation (see the middle cells in memory GFP+ cells stained for Foxo1 in Figure 4C).

### Foxo1 DNA binding activity is suppressed in HIV-1 infected resting CD4+ T cells

**Figure 5:**
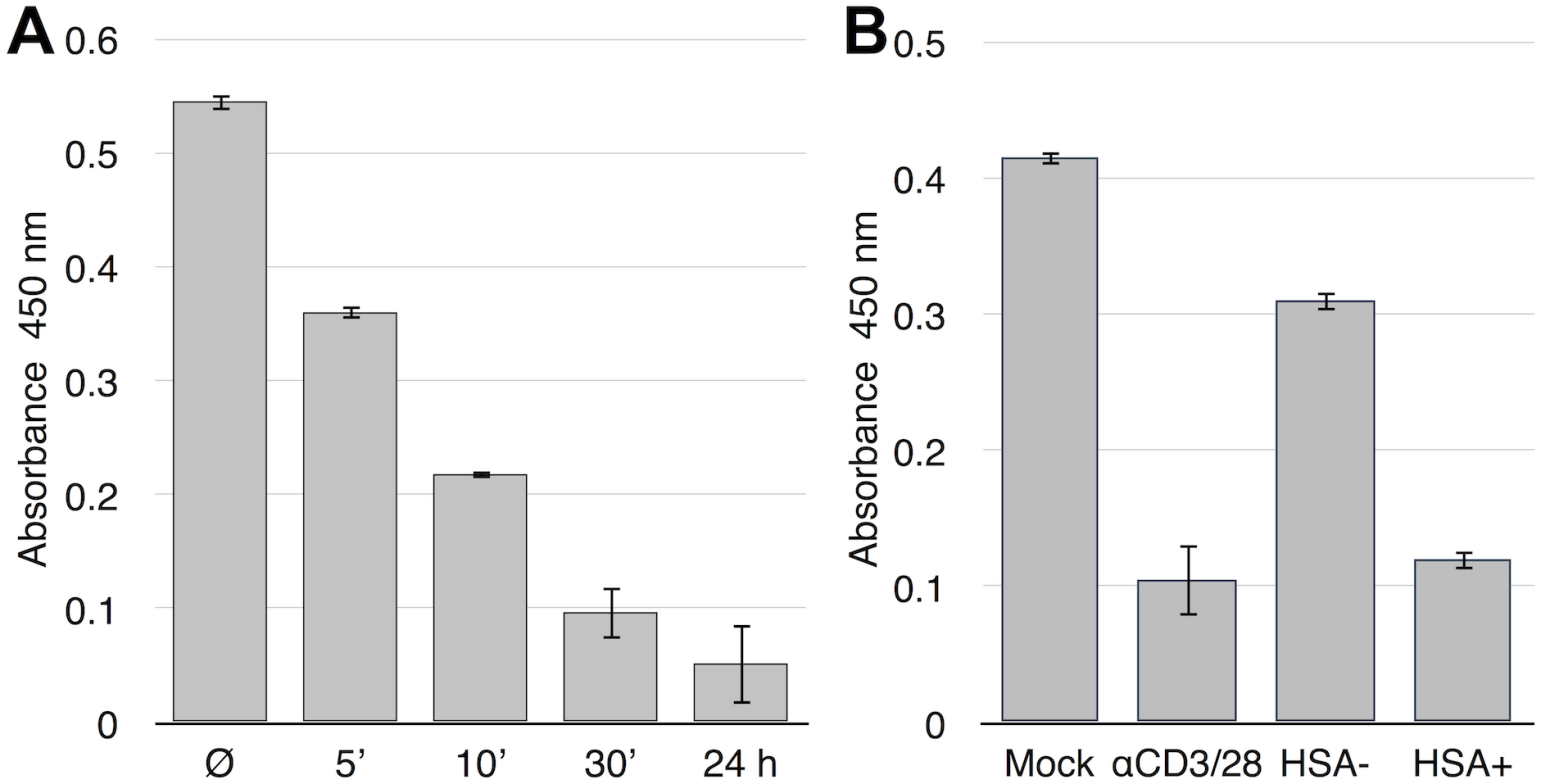
Foxo1 activity by TransAM ELISA. **A.** Time course of Foxo1 inactivation following T cell activation with anti-CD3/CD28 beads. The ability of nuclear Foxo1 to bind a target DNA oligonucleotide was analyzed using a TransAM Foxo1 kit (Active Motif). SD of triplicate analysis of each condition are shown from one representative experiment of 3. Specificity was demonstrated by successful competition with a free Foxo1 binding oligonucleotide but not with a negative control oligonucleotide (not shown). Error bars are SD of triplicate wells. **B.** Foxo1 activity in HIV-1 infected resting CD4 T cells, mock infected and beads activated cells. Cells were infected with HIV-1 HSA reporter virus for 7 days, then separated into HSA+ and HSA-negative subsets using an antibody to HSA and magnetic beads. Data are from one representative experiment of three independent experiments. Error bars are SD of triplicate wells.

We next directly examined nuclear Foxo1 activity using the TransAM transcription factor ELISA (Active Motif), which measures ability of Foxo1 to bind an oligonucleotide containing a Foxo1 target consensus sequence. We first performed a timecourse control experiment to examine the kinetics of Foxo1 inactivation following activation of resting T cells with anti-CD3/CD28 beads, observing that Foxo1 activity was progressively reduced in the first 24 hours after activation (Figure 5A). We then tested Foxo1 activity in HSA+ and HSA− cells, comparing them to mock infected or 24 hour beads-activated cells (Figure 5B). HSA− cells contained slightly less Foxo1 activity than mock control. Most importantly, in productively infected cells, Foxo1 activity was reduced to the low level observed in cells activated through the T cell receptor/coreceptor complex for 24 hours.

### A Foxo1 inhibitor accelerates HIV-1 replication

The question arises whether inactivation of Foxo1 confers a replicative advantage to HIV-1. The capacity of a cell to support viral replication is closely linked to its activation status, and Foxo1 is a primary suppressor of T cell activation. Therefore, we hypothesized that direct inhibition of Foxo1 might increase or accelerate HIV-1 replication. The Foxo1 inhibitor AS1842856 binds to Foxo1 and disables its ability to transactivate [78]. We first observed that AS1842856 did not interfere with nuclear Foxo1 binding to its DNA target sequence in the TransAM assay (not shown), consistent with a proposed mechanism of action in which AS1842856 does not interfere with DNA attachment but instead block unphosphorylated Foxo1 from binding to the its co-activator cAMP response element-binding protein [78].

**Figure 6:**
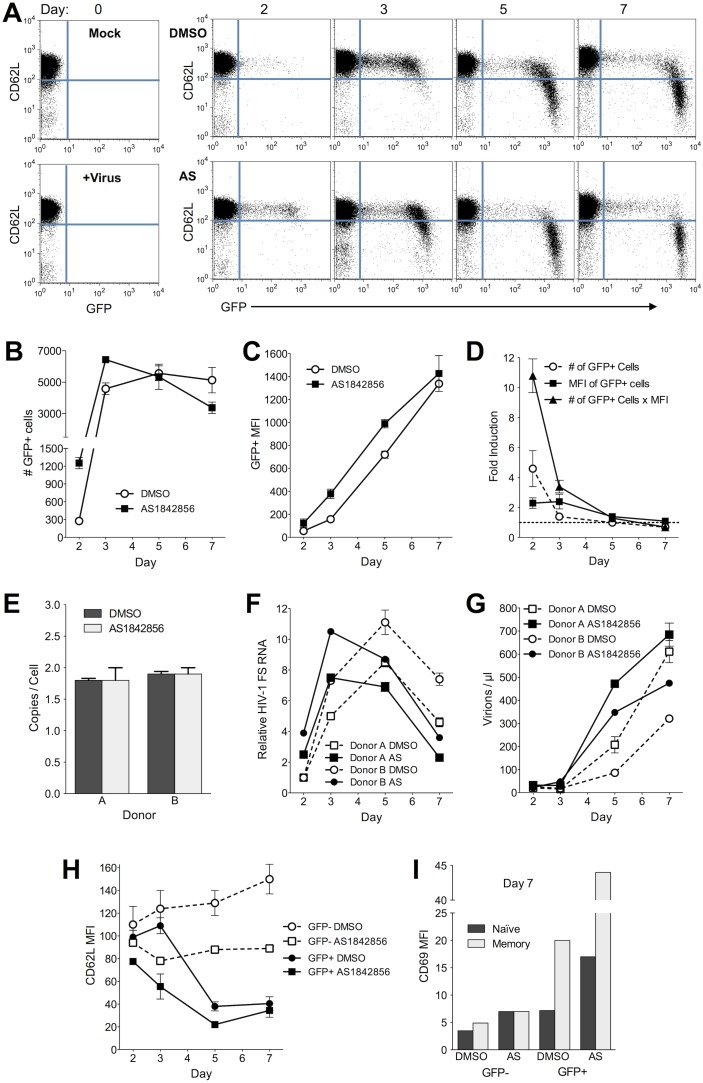
Foxo1 inhibitor AS1842856 accelerates HIV-1 expression. AS1842856 (AS) was applied to cells 1 day before and 1 day after infection with single round GFP reporter virus. CD45RO- naïve T cells are shown. **A.** GFP and CD62L expression in infected naïve CD4+ T cells on the indicated day after infection. Donor A is shown. **B.** The number of GFP+ cells on the indicated day after infection. Data are average and SD of the two donors. **C.** Mean fluorescence intensity (MFI) of GFP in the GFP+ cells. Data are average and SD of the two donors. **D.** Fold increase in the number and the MFI of GFP+ cells, and total GFP fluorescence in the cells calculated as the product of the number of GFP+ cells multiplied by their MFI. Data are average and SD of the two donors. **E.** Near-full length reverse transcripts on day 2 after infection for one representative cell donor. Data are averages and SD for replicate PCR reactions for each donor. **F.** HIV-1 fully spliced RNA presented relative to day 1 DMSO condition. Data are averages and SD for replicate PCR reactions for each donor. **G.** Virus production measured by genomic RNA in culture medium. Data are averages and SD for replicate PCR reactions for each donor. **H.** CD62L MFI on the indicated day. Data are MFI of GFP+ cells divided by background staining. Data are average and SD of the two donors. **I.** CD69 MFI on day 7 post infection on naïve and memory resting CD4+ T cells for donor A.

We applied AS1842856 to cells from two donors prior to and one day after infection with a single round pseudotyped virus. We then measured GFP, CD62L and CD69 expression, viral cDNA and RNA within the cells, and *de novo* virus output (Figure 6). GFP+ cells emerged earlier in the AS1842856 treated cultures, and at two days after infection, the total GFP fluorescence in the culture was enhanced by an order of magnitude, consistent with the interpretation that Foxo1 suppression assists the establishment of infection in cells (Figure 6A–D). Reverse transcription was unaffected by AS1842856 (Figure 6E), but the production of HIV-1 RNA was accelerated (Figure 6F). Interestingly, AS1842856 did not increase the maximum RNA production from the cells but only accelerated the achievement of this peak. Application of AS1842856 to sorted GFP+ cells that had reached their maximum expression had little effect, increasing GFP fluorescence 16% after 3 days (Figure S3 in File S1). CD62L was modestly down-modulated in the GFP-negative (Figure 6A, 6H) and control uninfected cells by a single AS1842856 dosing on day 0 (not shown), which when left untreated increased their CD62L expression over time. GFP+ AS1842856 treated cells down-modulated CD62L more rapidly than untreated cells in parallel with accelerated HIV-1 expression. Repeated dosing with AS1842856 over 9 days resulted in down modulation similar to HIV-1 infection (not shown). AS1842856 synergized with HIV-1 in productively infected cells to increase CD69 expression (Figure 6I), especially in memory cells, indicating early T cell activation. There are no Foxo1 or KLF2 consensus binding sequences in this HIV-1 strain, so these transcription factors are unlikely to be enhancing virus expression through direct binding to the viral promoter.

## Discussion

The suppression of Foxo1 activity of both infected resting naïve and memory CD4 T cells is a hitherto unknown dysregulation of cells by HIV-1. Foxo1 regulates fundamental properties of T cells, including their activation state and expression of a series of receptors controlling T cell migration such as CD62L; consequently the implications of this finding are wide ranging. The activation state of productively infected resting CD4 T cells was elevated, as they displayed increased CD69 and cyclin D2 expression and down-modulation of CD62L, all of which are Foxo1 target genes. A primary implication of this finding is that by raising the activation state of the infected cell, Foxo1 inactivation might enhance virus replication. Indeed, application of the Foxo1 inhibitor AS1842856 accelerated virus expression. Interestingly, the herpesvirus Epstein-Barr Virus also suppresses Foxo1 activity [79], suggesting that Foxo1 inactivation might not be an unusual viral strategy. Foxo1 is dysregulated in many cancers and the possibility of its therapeutic manipulation is avidly investigated [80]. Control of Foxo1 activity might also provide a means to activate or suppress HIV-1 expression in resting T cells.

We demonstrated Foxo1 inactivation in productively infected cells by both functional assays (DNA binding) and by ImageStream analysis showing that Foxo1 relocated to the cytoplasm in productively infected naïve and memory CD4 T cells. We confirmed this result by Western blot (unpublished data). Cytoplasmic Foxo1 can be degraded in a ubiquitin-dependent pathway [35], [50], [81], however we found that the total Foxo1 protein staining as well as its mRNA were nearly equal in cells with and without HIV-1 gene expression, indicating that Foxo1 inactivation by HIV-1 did not entail a reduction in Foxo1 protein or necessarily its transcription. The best characterized mechanism for Foxo1 inactivation is through phosphorylation, primarily by Akt (protein kinase B) that is activated in a PI3K dependent pathway following T cell receptor ligation, though phosphorylation by SGK (serum and glucocorticoid-induced kinase), casein kinase 1 and Cdk2 as well as acetylation and ubiquitinization can inhibit Foxo1 activity [82]. The ability of PI3K inhibitors to partially inhibit CD62L down-modulation in infected cells, plus the relocalization of Foxo1 to the cytoplasm both implicate phosphorylation in HIV-1-induced Foxo1 inactivation. HIV-1 is known to activate PI3K and Akt by the interaction of envelope with resting T cells [83] and through the activities of Tat and Nef [26], [84]. Each of these proteins has long been known to enhance both T cell activation [85]–[89] and infection of resting T cells [25], [29]. Our findings likely add another consequence of HIV-1-induced Akt activation; however, Foxo1 phosphorylation has been difficult to document in our system. Studies are underway to define the kinetics of Foxo1 inactivation and the HIV-1 gene(s) and signaling pathway(s) responsible.

PI3K is an upstream activator of Akt, which phosphorylates and inactivates Foxo1. Akt activation is an important mechanism to promote cell survival [90], [91], in part by reducing activity of Foxo1 and the related Foxo3a transcription factors. Inhibitors of PI3K and Akt are under development to treat cancers and certain inflammatory and infectious diseases [91]–[95]. HIV-1 Tat protein activates Akt in infected macrophages, providing a cytoprotective function; thus it has been proposed that Akt inhibition could provide a way to specifically target infected macrophages for death [96]–[98]. It may be that Foxo1 links HIV-1 infection and Akt activity with various sequella of infection to provide a therapeutically useful target for pharmacologic development.

CD62L down-modulation did not occur rapidly, but instead required several days of HIV-1 gene expression (Figure 6H) or treatment with Foxo1 inhibitor (unpublished data). These kinetics are similar to the genetic down-modulation that occurs following T cell activation, except that we did not observe the initial protease cleavage step following infection that has been reported by others [72], [73]. The half-life of CD62L mRNA is long [33], [55] and our unpublished data indicate that CD62L persists on the resting T cell surface for several days, so further studies will be required to reveal the kinetics of Foxo1, KLF2 and CD62L mRNA suppression after infection. Since *de novo* HIV-1 expression was required for these effects, there will of necessity be some delay between infection and Foxo1 suppression.

We hypothesized that the inhibition of Foxo1 activity with AS1842856 would increase or accelerate viral replication, and indeed, AS1842856 accelerated HIV-1 RNA transcription, but not reverse transcription. It was interesting that the peak of RNA production was similar between AS1842856 treated and control infections, consistent with the notion that Foxo1 inhibition mainly accelerates the establishment of infection. Also consistent with this interpretation was the observation that AS1842856 only modestly increased GFP fluorescence of cells that had been infected several days prior to treatment. As stated above, we find that daily dosing of uninfected CD4 T cells with AS1842856 results in much more complete CD62L down-modulation than the single dose we employed to assist HIV-1 infection (unpublished findings), consistent with the long half-life of CD62L mRNA and with the proposed mechanism of AS1842856 action [78].

Besides enhancing virus output, what are likely consequences of the reprogramming of infected naïve and memory resting T cells via Foxo1 suppression? Each of the genes that were up or down regulated can have a profound effect on the behavior of the cells, particularly their migration, activation and survival. These potential effects might be moot if the cell were to rapidly die due to viral cytopathicity, but resting T cells survive for much greater lengths of time than activated T cells [1], [22]. As previously mentioned, resting CD4 T cells are the dominant target of infection soon after transmission, the period when antiviral immunity is being established that will determine the outcome of infection [7]. Much of the latent reservoir is also established early in infection [99], [100]. By manipulating the cells’ activation state and the expression of receptors that support migration in and out of lymphoid tissues (and which also possess intracellular and extracellular signaling properties), HIV-1 could interfere with the establishment of antiviral immunity through suppression of Foxo1 activity.

The overall pattern of gene regulation in the productively infected naïve CD4 T cells was consistent with the observed suppression of Foxo1 activity by HIV-1. Although naïve T cells are a minority of infected T cells *in vivo*, they are important targets of infection [2], [3], [101], [102]. We chose to examine gene regulation in this population because of its uniform expression of CD62L, whereas memory cells are heterogeneous in CD62L expression, with many T_EM_ expressing no CD62L. Future analysis of TCM will be useful in order to more fully understand Foxo1 dysregulation by HIV-1, and preliminary studies indicate a pattern of gene regulation by HIV-1 similar to naïve cells (unpublished data). One of the genes that Foxo1 maintains in resting T cells is KLF2, a second master regulator of T cell activation and migration [60], [103], [104], and we observed that KLF2 mRNA was strongly suppressed in infected cells. Prior studies have demonstrated that ectopic expression of KLF2 in proliferating and activated cells returns them to a quiescent state, and loss of KLF2 expression has been shown to result in increased T cell activation [57], [105]. KLF2−/− mice lack naïve T cells in lymphoid organs, acquiring the homing characteristics of activated T cells [105]. In the present study the mRNA of several genes that are known to be transcriptionally activated by Foxo1 and/or KLF2 were lower in productively infected cells, including CD62L, IL-7rα, Fam65B and S1P_1_, CD52, Myc and CCR5 [56], [60], [62], [103], [105], [106]. mRNA for two genes that Foxo1 suppresses, CD69 and Cyclin D2, were increased in infected cells, again consistent with Foxo1 inactivation. Interestingly, the Foxo1-mediated suppression of Cyclin D2 transcription does not require Foxo1 DNA binding activity [107], evidence of Foxo1 gene regulation through interaction with protein binding partners [47]. Myc has been shown to suppress HIV-1 transcription [108] so its suppression in productively infected cells is quite interesting.

CD62L, KLF2, CD69, S1P_1_, CCR5 and Fam65b are each involved in T cell migration. While CD62L is required for entry into lymph nodes, S1P_1_ is required for egress in response to its ligand S1P present in blood [104], [109]. KLF2 and Foxo1 both induce S1P_1_ expression [45], [60], [62]. T cell responses to S1P_1_ are impaired in HIV-1 infected individuals, perhaps contributing to lymphadenopathy [110]. S1P_1_ and CD69 are mutual antagonists [110], [111] and CD69 expression inhibits S1P_1_-dependent egress from lymphoid organs until later in the activation cascade, when CD69 levels decline [112]. CD69 also regulates chemokine expression and T cell accumulation in the intestine [113]. Thus, by increasing CD69 and suppressing both CD62L and S1P_1_, HIV-1 may disrupt both lymphoid tissue entry and exit functions of infected cells. Tat has been shown to induce CD69 expression through NF-κB activation [114]. However, we saw no NF-κB nuclear translocation, so Foxo1 suppression seems to be the operative in our system. The increase in CD69 which we observed was rather modest compared with the levels achieved following T cell receptor stimulation (Figure 1), and whether S1P_1_ is being suppressed by CD69 binding to S1P_1_ [115], [116] awaits further study.

The mRNA of cell cycle regulator p21^CIP1^ was also higher in productively infected cells. This finding is interesting in light of the findings that Foxo proteins are activators of p21^CIP1^ transcription in neural cells [117] and that KLF2 upregulates p21 transcription in Jurkat T cells [118]. Based on these findings, we would expect lower p21 mRNA levels in HIV-1 infected cells. Our contrary finding may reflect cell-type specific differences or the possibility that transformed cells do not reproduce the regulatory pathways of primary cells. On the other hand, Myc was repressed in Foxo−/− mice [119], and Seoane et al. [117] also reported that Myc represses p21, so in this paradigm Myc repression in Foxo1-suppressed infected cells would result in increased p21 – the outcome we observed. To make the situation more complicated, overexpression of KLF2 represses Myc transcription in Jurkat cells [60], [120], again, a finding hard to reconcile with our data; but, in a further apparent contradiction, a study KLF2−/− mice found no alteration in Myc in CD8 T cells [57] reviewed in [31]. Finally, HIV-1 Vpr can induce p21 transcription [121]–[123]. In macrophages, this p21 upregulation enhanced virus replication [123]. Studies are underway examining the contribution of Vpr to cellular gene regulation in resting T cells.

CCR7, Foxo1 and Foxo3a are also known transcriptional targets of Foxo1 or Foxo1 plus KLF2, and their mRNA levels remained unchanged during productive infection. Why expression of these genes was relatively unchanged remains to be investigated. Although CCR7 mRNA remained constant, CCR7 receptor staining was modestly reduced on productively infected cells, consistent with a recent report that Vpu modestly down-modulates CCR7 surface expression in activated T cells [67]. Here we report that HIV-1 modestly down-modulates CCR7 in resting T cells while not altering CCR7 mRNA levels.

The suppression of CCR5 mRNA in GFP+ bright cells was consistent with recent findings that KLF2 activated CCR5 expression and bound to the CCR5 promoter in resting T cells [61], but both of these reports are inconsistent with a study in KLF2 knockout mice [105]. CCR5 is the ligand for chemokines CCL3 and CCL4 (MIP1α and β) and its suppression would be expected to alter homing to inflammatory sites. Another migration-related gene suppressed in infected cells was Fam65b. Fam65b negatively regulates RhoA activity and chemokine-induced migration [124], and its transcription is activated in T cells by Foxo1 [64]. Together, the multitude of disruptions by Foxo1 suppression make proper homing to lymphoid tissues and effector sites appear unlikely, though functional tests will be required. A recent study found that quite low levels of KLF2 were sufficient to maintain CCR5 expression and infectability [61], and our data reveal exceptionally strong suppression of CCR5 mRNA in the GFP bright cells. HIV-1 Nef is known to down-modulate CCR5 [125], [126], and the present work demonstrates a second mechanism (transcriptional repression) by which HIV-1 may reduce CCR5 in resting CD4 T cells. CCR5 receptor expression on resting CD4 T cells is difficult to quantify and further studies are underway. On the other hand, we did observe efficient down-modulation of CD4 surface expression in resting T cells (Figure 1) as well as Class I MHC down-modulation (unpublished data), but neither of their mRNA levels were affected by infection. GFP+ dim cells had about 1.5 times more CCR5 mRNA than the GFP− cells, which could be either preferential infection of cells that are slightly more activated or active upregulation in this subset. GFP+ dim cells display only early HIV-1 gene expression [22], [127], so perhaps and early gene such as Tat or Nef increases CCR5 mRNA, while late HIV-1 gene expression is necessary for KLF2 suppression. It is interesting to note that in addition to binding to vascular endothelium, CD62L also seems important for regulating expression of the chemokine receptor CXCR4, the other HIV-1 co-receptor besides CCR5 [128]. Thus the potential for HIV-1 to disrupt cell trafficking via suppression of Foxo1 activity would appear to be wide ranging.

Finally, in light of the ability of HIV-1 to down-modulate CD62L, the use of CD62L to discriminate between central memory T cells and effector memory T cells during HIV-1 infection may be problematic. Our findings demonstrate that infected TCM (CD45RA-RO+CD62L+) that have down-modulated CD62L can, superficially at least, masquerade as infected T_EM_ (CD45RA-RO+CD62L−) in flow cytometry analysis. Additionally, TCM uniformly express CD62L, while TEM are sometimes represented as being uniformly CD62L-negative [129]. CCR7-negative T_EM_ are actually heterogeneous in their expression of CD62L (Figure S2 in File S1, [130]), so CCR7 expression or any of several other receptors [130] should be more useful markers for T_CM_ vs. T_EM_ discrimination.

## Materials and Methods

### Cells

Peripheral blood CD4+ T cells from healthy HIV-1-negative donors were isolated by negative selection using the Dynabeads Untouched Human CD4 T cell magnetic separation Kit (Life Technologies) per manufacturer direction as previously described [22]. Purified cells were routinely ≥99% small, CD4−, CD25−, CD38−,CD69−, and HLA-DR- quiescent T cells [22]. Blood was purchased from the New York Blood Center as de-identified samples. No protected health information was collected. The New York University Committee on Activities Involving Human Subjects (UCAIHS) determined that these samples do not constitute human research. Purified CD4+ T cells were cultured at 2×10∧6 cells per ml in Gibco Advanced RPMI 1640 with 10% fetal bovine serum (HyClone) plus penicillin and streptomycin (Gibco) and 50 µM β-mercaptoethanol (Sigma). Where indicated, IL-7 (2 ng/ml, BioVision) or IL-4 (25 ng/ml) (R&D Systems) was added to the culture medium. Where indicated, cells were activated with Dynabeads Human T-Activator CD3/CD28 (Life Technologies), as per the manufacturer's instructions.

### Viruses

Virus construction has been described previously [22], [127], [131]. NLENG1-IRES (Fig. 1, 2) is a replication competent HIV-1 NL4-3 backbone GFP reporter virus, identical to NLENY1-IRES and NLENC1-IRES [131] except that it contains the eGFP gene (Clontech) in place of eYFP or eCFP. Infections with this virus were restricted to a single round of infection by treatment with the protease inhibitor indinavir (2 µM). NLENG1-ESI (Figures 3, 4, 6) is an envelope mutant HIV-1 GFP reporter virus previously described [22], [127]. NLENHSA-ESI (Figure 5) is an HIV-1 NL4-3 backbone HSA (mCD24) reporter virus constructed identically to NLENG1-ESI, except that it contains the gene for murine CD24 (Heat Stable Antigen – HSA) in place of GFP.

### Infections

The titers of the virus stocks were determined using TaqMan qRT-PCR for HIV-1 RNA (target in integrase) and normalized to a nominal 400 virion equivalents per cell for infection. Infections were performed by spinoculation in the presence of 5 µg/ml DEAE dextran (Sigma) for 2 h at 1,200×g and 37°C [22], [132]. Cells were then washed and plated. For FKHR TransAM experiments, IL-7 treated CD4 T cells were infected with NLENHSA-ESI. At 7 dpi., cells were ficolled in order to remove dead cells, then stained with a phycoerythrin (PE) labeled anti-mCD24 and bound to anti-PE magnetic microbeads (Miltenyi Biotec). Positive and negative cells were separated using the MACS system (Miltenyi Biotec). Purity was routinely >95%.

Cell treatments included PMA (2 nM, Fisher Scientific), LY294002 (5 µM, BioVision), PI-103 (5 µM, EMD Millipore), TAPI-1 (50 µM, Santa Cruz Biotechnology). Non-nucleoside reverse-transcriptase inhibitor Efavirenz (2 µg/ml) and Protease inhibitor Indinavir (2 µM) were obtained from the NIH AIDS Research and Reference Reagent Program. The Foxo1 inhibitor 5-amino-7-(cyclohexylamino)-1-ethyl-6-fluoro-4-oxo-1,4-dihydroquinoline-3-carboxylic acid (AS1842856) [78], was purchased from EMD Millipore and used at a final concentration of 50 nM. DMSO controls were performed with a corresponding dilution of 1 in 20,000.

#### Tonsil cell infections

Tonsils were obtained as de-identified specimens from discarded pathologic specimens of children without known HIV-1 infection undergoing elective tonsillectomies at Children’s Hospital Denver in accordance with the Colorado Multiple Institutional Review Board (CMIRB), which reviewed this protocol on September 23, 2008 and determined that it does not constitute human subjects research. For this reason, informed consent is not required.

Tonsils were mechanically disaggregated in sterile phosphate buffered saline. The cell suspension was filtered through a 70 micron filter (Fisher Scientific, Denver, CO) and washed with PBS. Single cell suspensions were infected with NLENG1-IRES as described [133], then IL-4 (25 ng/ml) was added to the cultures and again on day 3 post infection. For flow cytometry, cells were stained with anti-CD3-PerCP, anti-CD8-PerCP-Cy5, anti-CD45RA-PE-Cy5, anti-CD62L-PE, (all from BD Pharmingen) and analyzed on a Becton Dickinson (BD) LSR II flow cytometer (UC Denver). Gating for GFP expression was on CD3+CD8^–^CD45RA+ cells.

### Flow Cytometry

Flow cytometry was performed on a BD FACSort flow cytometer upgraded by Cytek Development, Fremont, CA, to contain 488-nm, 407-nm, and 637-nm lasers and 5 fluorescence detectors. Enhanced Green fluorescent protein (GFP) was detected in FL-1 using 510/21-nm and a 540-nm short-pass dichroic splitter. Compensation was applied during data collection on the basis of single-color controls. Data collection was with CellQuest Pro software for Mac OS X (BD Biosciences). Flow data were analyzed using FlowJo (v9 and v10) software for Mac OS X (Tree Star). T(x) population comoparison analysis was with FlowJo 9 [134]. Proliferation analysis was performed using eFluor670 (eBioscience). CD4 (PerCP-Cy5.5, SK3) and HLA-A2 (PerCP-Cy5.5, BB7.2) antibodies were purchased from BD Pharmingen. Memory and Naïve CD4 T cells were identified by positive CD45RA staining and/or negative CD45RO staining. CD45RA (Pacific Blue, HI100), CD45RO (Pacific Blue, UCHL1), CD62L (PerCP-Cy5.5 and APC, DREG-57), CD69 (PerCP-Cy5.5, FN50), and CCR7 (APC, TG8/CCR7) antibodies were purchased from BioLegend. Phosphatidylserine externalization was detected with Annexin-V Pacific Blue (Life Technologies) and cellular membrane permeability was assessed by 7-AAD (7-Aminoactinomycin D) (Life Technologies) incorporation. Cell sorting was performed using a BD FACSAria flow cytometer at the New York University Medical Center Flow Cytometry and Cell Sorting Center.

### ImageStream

Foxo1 rabbit antibody (C59H4) was purchased from Cell Signaling and NF-κB (p65) rabbit antibody (C-20) was from Santa Cruz Biotechnology. CD45RO antibody was from BioLegend. Staining were performed following Cell Signaling protocol for intracellular staining, with modifications. Briefly, T cells were harvested at the indicated times and fixed with 2% paraformaldehyde (PFA) for 10 min at 37°C. Cells were then chilled on ice for 1 min and mixed while vortexing with 9 volumes of ice cold 100% methanol to a final concentration of 90% for permeabilization. Cells were incubated for 1 hour at −20°C and washed with incubation buffer (0.5% BSA in PBS). Cells were blocked in incubation buffer for 10 min. Staining were performed for either transcription factor plus mouse anti-CD45RO PerCP-Cy5.5 (CD45RO staining was performed after permeabilization to avoid fluorochrome degradation in methanol). Cells were incubated for 1 hour at room temperature, washed with 0.5% BSA PBS and stained with anti-rabbit DyLight 649 (BioLegend). Cells were incubated another hour at room temperature then washed. Prior to acquisition, cells were incubated at 10∧6 cells per ml in DAPI staining solution (Life Technologies) for 15 min. Cells were then pelleted and resuspended in the same solution at 30×10∧6 cells per ml for ImageStream acquisition. Acquisition was performed in the Flow Cytometry Resource Center at Rockefeller University (New York) using Amnis ImageStream-X and analysis was done with the Amnis IDEAS software and FlowJo (v10).

Single-color controls were acquired in order to construct a compensation matrix that was applied to all experimental samples. Similarity score between each transcription factor (Foxo1 or NF-κB) and DNA staining with DAPI was calculated using IDEAS software Similarity Feature. which is the log-transformed Pearson’s correlation coefficient between the pixel values of two image pairs. The degree of nuclear localization of a protein is measured by the pixel intensity correlation between staining for that protein and DNA staining [135], [136]. The higher the Similarity Score, the higher the level of nuclear localization.

### Foxo1 TransAM

Foxo1 (FKHR) activity was quantified using the FKHR TransAM ELISA system from Active Motif. Briefly, T cells were treated and harvested as described in “Infections”, then nuclear proteins were extracted using the Active Motif Nuclear Extraction Kit. Nuclear proteins were quantified with the NanoOrange Protein Quantitation Kit (Life Technologies). Samples were tested in triplicate wells of 3 µg total proteins per replicate. Foxo1 DNA binding activity to an oligonucleotide containing the Foxo1 target consensus sequence (5′-TTGTTTAC-3′) was measured following the Active Motif’s instructions. Foxo1 binding specificity was verified by adding competitive specific or scrambled oligonucleotides provided in the kit. ELISA plate O.D. was read on a Molecular Devices SpectraMax M5 spectrophotometer with detection of absorbance at 450 nm and a reference wavelength of 655 nm.

### Quantification of HIV-1 DNA

qPCR methods have been described previously [22]. Briefly, DNA from cells infected with benzonase-treated viruses was purified using a DNeasy Blood & Tissue Minikit or an AllPrep Minikit (Qiagen) with RNase A (Sigma) digestion. Quantitative real-time PCR for DNA analysis was performed using a QuantiTect Probe PCR kit (Qiagen). Primers and TaqMan probes were purchased from Integrated DNA Technologies (IDT). Amplification and detection were performed on a Chromo4 real-time PCR machine (Bio-Rad), and the data were analyzed using the manufacturer's Opticon Monitor 3 software.

Quantification of total HIV DNA was performed using:

forward primer: ZXF F (5′-AAGTAGTGTGTGCCCGTCTGT-3′),

reverse primer: ZXF R (5′-GCTTCAGCAAGCCGAGTC-3′),

and probe: ZXF (5′-56-FAM-TGTGACTCT-ZEN-GGTAACTAGAGATCCCTCAGACCC-3IABlk_FQ-3′,

where FAM is 6-carboxyfluorescein, ZEN is an internal quencher (IDT), and 3IABlk_FQ is the 3′ Iowa black FQ quencher. Quantification of 2-LTR circles [22] was performed using:

forward primer: 2L-f2 (5′-TGTTGTGTGACTCTGGTAACTAGAGATCCC-3′),

reverse primer: 2L-r2 (5′-GATATCTGATCCCTGGCCCTGG-3′),

probe: 2L-z2 (5′-56-FAM-CCACACACAAGGCTACTTCCCTGATTGGCAG-3IABlk_FQ-3′).

CD4 gene DNA amplification (see primers in the qRT-PCR section) was used for normalization of the total number of HIV-1 DNA copies per cell. Standardization for quantification of total HIV-1 DNA was performed with 10-fold serial dilutions of genomic DNA extracted from Jurkat cells containing 2 integrated HIV-1 NL4-3 proviruses.

DNA qPCR used the following thermal cycling program: step 1: 95°C for 15 min; step 2: 94°C for 15 sec; step 3: 60°C for 30 sec; step 4: fluorescence data collection; and step 5: return to step 2×40.

### qRT-PCR

RNA was isolated from virus-containing culture medium or infected cells using an RNeasy Minikit (Qiagen) with on-column DNase digestion (40 min). Quantitative real-time RT-PCR for RNA was performed using a QuantiTect Probe RT-PCR kit (Qiagen). All qRT-PCR used the following thermal cycling program: step 1: 50°C for 30 min; step 2: 94°C for 15 min; step 3: 94°C for 15 sec; step 4: 60°C for 30 sec; step 5: fluorescence data collection; and step 6: return to step 3×40.

#### Quantification of HIV-1 in supernatants (virus stock and experimental output)

We used primers and TaqMan probes specific for a shared region of integrase RNA sequence. Quantification of HIV-1 RNA was performed against serial 10-fold dilutions of a synthetic RNA molecule containing the integrase target (67). Forward primer : FL-U Sense (5′-CAATTTCACCAGTACTACAGTT-3′),

reverse primer: FL Sense (5′-GAATGCCAAATTCCTGCTTGA-3′) (79),

probe: FL-U (5′-56-FAM-AAGGCCGCC-ZEN-TGTTGGTGGGCG-3IABlk_FQ-3′).

Control assays for DNA carryover were routinely performed and were either completely negative or contained ≤1/40,000 RNA molecules.

#### Quantification of fully spliced HIV RNA in infected cells

We used primers and TaqMan probes (Integrated DNA Technologies) detecting fully spliced RNA specific for NL4-3 HIV. Quantification of FS HIV RNA was performed against serial 10-fold dilutions of a synthetic RNA molecule containing the fully spliced (FS) target.

Forward primer: DNL287 (5′-GGAGACAGCGACGAAGAGC-3′),

reverse primer: DNL288 (5′-CTCTCCACCTTCTTCTTCTATTCC-3′),

probe: LA45-LA41: (5′-56-FAM-CATCAGAACAGTCAGACTCATCAAGCTTCTCTATCAAAG-3IABlk_FQ-3′).

#### Quantification of cellular mRNA

With the exception of CD4 primers, which were from IDT, primers and TaqMan probes for mRNA quantification were from Life Technologies (Table S1 in File S1).

CD4 RNA the primers and probes:

Forward primer: CD4F2 Sense (5′- GCCAACTCTGACACCCAC-3′),

reverse primer: CD4R2 Sense (5′- GACTCCTACATTGCACTGAG-3′),

probe: CD4P2 (5′-56-FAM-CAGAGCCTG-ZEN-ACCCTGACCTTGGAGA-3IABlk_FQ-3′).

Relative expression of cellular mRNAs in infected T cell subsets (Fig. 3C and D) was analyzed using the Comparative Ct method [137] and normalized to the average Ct of 2 endogenous housekeeping genes RPL13A and IPO8 [138]. Results were expressed in fold inductions in comparison to the reference sample of sorted GFP negative T cells.

Fold induction = 2^−ΔΔCt^.

ΔΔCt = ΔCt of sample–ΔCt of reference.

ΔCt = Ct of gene of interest–average Ct of house keeping genes.

## Supporting Information

File S1 **File S1 includes the following: Figure S1. CD62L expression is maintained in the presence of common gamma chain cytokines.** Resting CD4+ T cells were cultured with no cytokine (Ø) or the indicated cytokine for 2 days then analyzed for CD45RA and CD62L expression by flow cytometry. Neg. control was with an isotype-matched non-specific antibody. IL-2 (50 u/ml), IL-4 (25 ng/ml), IL-7 (2 ng/ml), IL-15 (10 ng/ml). Data are representative of >3 independent experiments. **Figure S2. CD62L is down-modulated in HIV-1 expressing cells in both central memory and effector memory CD4+ T cells, and CCR7 is modestly down-modulated.** IL-7-treated resting CD4+ T cells were infected with a single round GFP virus and then analyzed as in Figure 1, with additional staining with CCR7 to distinguish central memory (T_CM_) and effector memory (T_EM_) cells. Data are representative of >3 independent experiments. **A.** CD62L is down-modulated on naïve (CD45RA+), central memory (TCM, CD45RA-CCR7+) and effector memory (T_EM_, CD45RA-CCR7−) resting CD4+ T cells. A subset of TEM naturally lacks CD62L expression. **B.** CCR7 is slightly down-modulated on naïve and memory CD4+ T cells expressing HIV-1. Data are representative of >5 experiments which consistently show 20%–30% loss of CCR7 mean fluorescence intensity (MFI) in the HIV-1 expressing (GFP+) cells. Grey: isotype-matched IgG control antibody staining. Other histograms are color coded to match the legend font color. **C.** HIV-1 expression is highest in effector memory T cells as measured by GFP MFI. **Figure S3. Foxo1 inhibitor AS1842856 applied to productively infected GFP+ cells.** IL-7 treated resting CD4+ T cells were infected with a single round env-pseudotyped HIV-1 GFP reporter virus and sorted for GFP+ cells. On day 17 after infection, AS1842856 was applied and GFP expression was analyzed 3 days later. Cell viability was 14–20%. **Table S1.** TaqMan primer and probe sets for quantification of cellular RNA in Figure 3. **Table S2.** Values for RNA expression graphed in Figure 3. HIV-1 FS is fully spliced Rev-independent early viral RNA. HIV-1 FL is full length Rev-dependent late viral RNA.
(PDF)
