# Supplementary material for: Suppression of Foxo1 Activity and Down-Modulation of CD62L (L-Selectin) in HIV-1 Infected Resting CD4 T Cells
Source: PLoS One. 2014 Oct 16;9(10):e110719. doi: 10.1371/journal.pone.0110719 (PMC4199762; doi:10.1371/journal.pone.0110719)
Supplement: File S1 — includes the following: Figure S1. CD62L expression is maintained in the presence of common gamma chain cytokines. Resting CD4+ T cells were cultured with no cytokine (Ø) or the indicated cytokine for 2 days then analyzed for CD45RA and CD62L expression by flow cytometry. Neg. control was with an isotype-matched non-specific antibody. IL-2 (50 u/ml), IL-4 (25 ng/ml), IL-7 (2 ng/ml), IL-15 (10 ng/ml). Data are representative of >3 independent experiments. Figure S2. CD62L is down-modulated in HIV-1 expressing cells in both central memory and effector memory CD4+ T cells, and CCR7 is modestly down-modulated. IL-7-treated resting CD4+ T cells were infected with a single round GFP virus and then analyzed as in Figure 1, with additional staining with CCR7 to distinguish central memory (TCM) and effector memory (TEM) cells. Data are representative of >3 independent experiments. A. CD62L is down-modulated on naïve (CD45RA+), central memory (TCM, CD45RA-CCR7+) and effector memory (TEM, CD45RA-CCR7−) resting CD4+ T cells. A subset of TEM naturally lacks CD62L expression. B. CCR7 is slightly down-modulated on naïve and memory CD4+ T cells expressing HIV-1. Data are representative of >5 experiments which consistently show 20%–30% loss of CCR7 mean fluorescence intensity (MFI) in the HIV-1 expressing (GFP+) cells. Grey: isotype-matched IgG control antibody staining. Other histograms are color coded to match the legend font color. C. HIV-1 expression is highest in effector memory T cells as measured by GFP MFI. Figure S3. Foxo1 inhibitor AS1842856 applied to productively infected GFP+ cells. IL-7 treated resting CD4+ T cells were infected with a single round env-pseudotyped HIV-1 GFP reporter virus and sorted for GFP+ cells. On day 17 after infection, AS1842856 was applied and GFP expression was analyzed 3 days later. Cell viability was 14–20%. Table S1. TaqMan primer and probe sets for quantification of cellular RNA in Figure 3. Table S2. Values for RNA expression graph [file pone.0110719.s001.pdf]

Figure S1

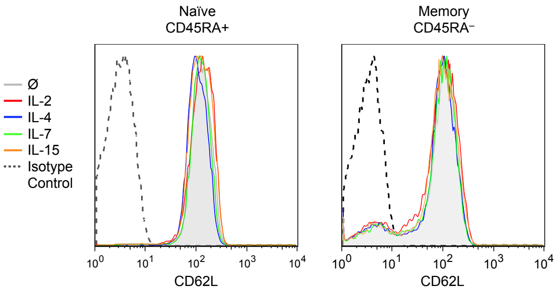

Naïve
CD45RA+
Memory
CD45RA⁻
Ø
IL-2
IL-4
IL-7
IL-15
Isotype
Control
CD62L
CD62L

Figure S2

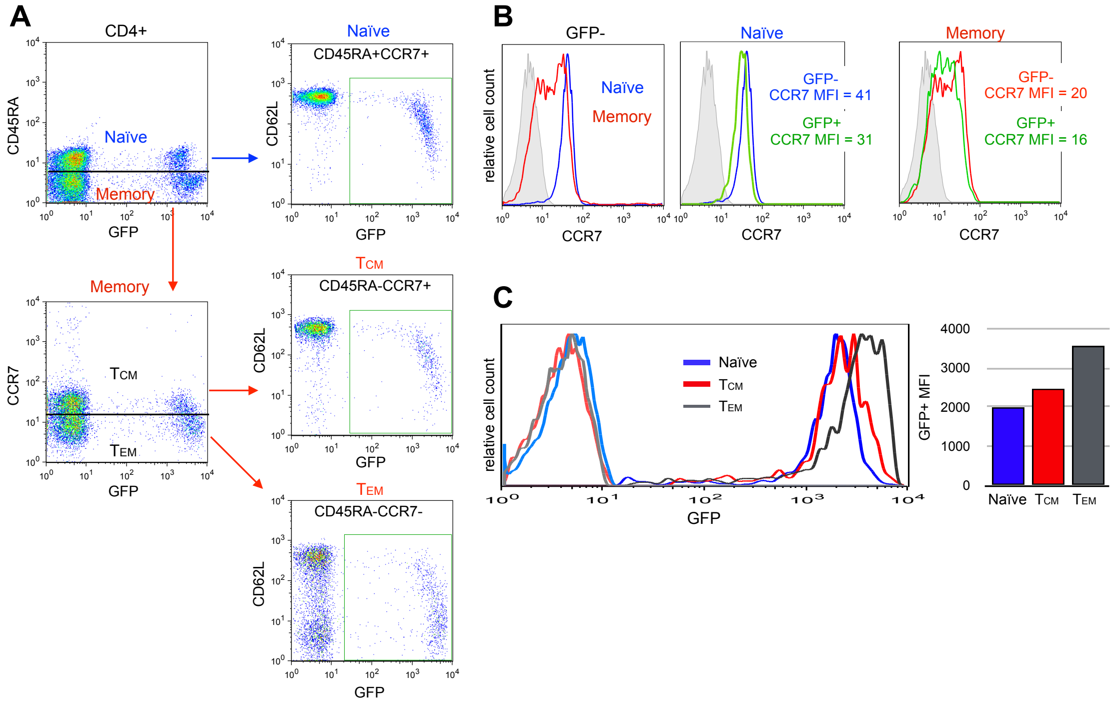

A
CD4+
CD45RA
GFP
Naïve
Memory
CCR7
TCM
TEM
CD45RA+CCR7+
CD45RA-CCR7+
CD45RA-CCR7-
CD62L
B
GFP-
relative cell count
CCR7
GFP-
CCR7 MFI = 41
GFP+
CCR7 MFI = 31
GFP-
CCR7 MFI = 20
GFP+
CCR7 MFI = 16
C
GFP+ MFI
4000
3000
2000
1000
0

Figure S3

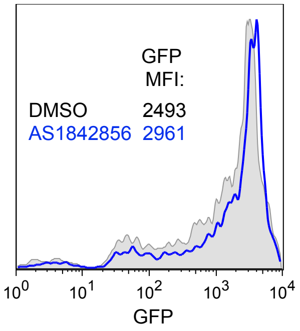

Table S1

Gene	a.k.a.	TaqMan Primer/Probe set	Company
Cyclin D2	CCND2	Hs00153380_m1	Life Technologies
CCR5	CD195	Hs99999149_s1	Life Technologies
CCR7	CD197	Hs01013469_m1	Life Technologies
CD4		see methods	Integrated DNA Tech.
CD52	CDW52, CAMPATH-1 Ag	Hs00174349_m1	Life Technologies
CD62L	SELL, L-selectin, LAM-1	Hs00174151_m1	Life Technologies
CD69		Hs00934033_m1	Life Technologies
Fam65b	C6orf32	Hs00210599_m1	Life Technologies
Foxo1	FKHR, FOXO1	Hs01054576_m1	Life Technologies
Foxo3a	FKHRL1, FOXO3	Hs00818121_m1	Life Technologies
HLA-A	Major Histocompatibility Complex, Class I, A	Hs01058806_g1	Life Technologies
IL-7Rα	IL-7 receptor alpha chain, CD127	Hs00902334_m1	Life Technologies
KLF2	Krüppel-like Factor 2, LKLF	Hs00360439_g1	Life Technologies
Myc	c-Myc	Hs00153408_m1	Life Technologies
$p21^{CIP1}$	WAF1, CDKN1A	Hs00355782_m1	Life Technologies
$S1P_1$	EDG1, S1PR1, CD363	Hs01922614_s1	Life Technologies
IPO8*	Importin 8	Hs00183533_m1	Life Technologies
RPL13A*	60S ribosomal protein L13a	Hs04194366_g1	Life Technologies

* housekeeping control genes

Table S2

Values graphed in Figure 3*				
Gene	GFP expression			
	−	+	++	+++
HIV-1 FS	1	225	447	546
HIV-1 FL	1.52	185	526	1254
KLF2	1	0.52	0.4	0.22
IL-7rα	1	0.41	0.37	0.21
Fam65b	1	0.71	0.62	0.49
CD62L	1	0.57	0.57	0.42
S1P1	1	0.63	0.66	0.47
CD52	1	0.68	0.59	0.4
Myc	1	0.62	0.27	0.23
CCR5	1	1.42	0.09	0.08
CCR7	1	0.89	1.07	1.14
Foxo1	1	0.89	0.83	0.76
Foxo3a	1	1.07	1.15	1.06
CD69	1	1.31	1.89	2.37
Cyclin D2	1	1.77	2.53	2.61
$p21^{CIP1}$	1	2.34	2.19	3.44
HLA-A	1	0.85	0.88	0.82
CD4	1	1.04	1.2	1.14

*Values are set relative to GFP-negative cells (=1) except HIV-1 full length RNA (FL), which is set relative to HIV-1 FS RNA in the GFP-negative cells.
